# Blueprint for safe transition from a low- to high-volume pancreatic surgery center

**DOI:** 10.1016/j.sopen.2022.09.006

**Published:** 2022-09-29

**Authors:** Edward S. Cho, Michael E. Zenilman, Paul H. McClelland, Daniel Rodriguez, Justin Steele, Bashar Fahoum, Michael Wayne

**Affiliations:** Department of Surgery, Weill Cornell Medicine, NewYork-Presbyterian Brooklyn Methodist Hospital, Brooklyn, NY 11215

## Abstract

We describe a straightforward model to implement a high volume specialty surgery program at a community hospital. Using pancreatic surgery as an example, we employed published processes in three arenas. First, mandatory multidisciplinary tumor board presentations captured all the patients considered for surgery. Then, perioperative protocols using tools such as enhanced recovery and teamwork in the perioperative arena created a reproducible and safe environment for complex surgery. We critically reviewed all complications using the Clavien-Dindo methodology, and confirmed our favorable outcomes via the targeted NSQIP program. These standard steps can be used for implementation of a new complex surgical procedure.

## INTRODUCTION AND METHODS

High-volume centers specializing in complex surgical procedures are associated with improved outcomes. This effect is seen across most surgical subspecialties, including pancreatic surgery [1].

Our institution underwent a rapid transition from an urban community hospital to an academic high-volume pancreatic surgery center. NewYork-Presbyterian Brooklyn Methodist Hospital (NYPBMH) is a 591-bed level 2 trauma center in the culturally diverse borough of Brooklyn. Before 2017, NYPBMH had mature services in interventional radiology, advanced endoscopy, surgical intensive care and operating rooms, but low volumes in pancreatic surgery. Pancreatic resections were performed by multiple general surgeons on a case-by-case basis. In late 2017, a dedicated team led by a fellowship trained hepatopancreatobiliary (HPB) surgeon was created. To adjust for this change, several pathways were effected to better care for patients undergoing pancreatic surgery (Table 1). A policy was created mandating that all patients be presented at our institution's multidisciplinary tumor board prior to any treatment. An integrated Enhanced Recovery After Surgery (ERAS) was implemented to standardize orders across the perioperative spectrum [2]. To increase safety in the operating room, all patients undergoing pancreatic resection were screened and risk-stratified by the anesthesiology service. High risk patients were flagged, alerting the perioperative and intensive care teams to the potential risk of blood loss. This system allowed for adequate preparation on the part of critical care providers for the management of complex patients undergoing major operations.

**Table 1:** Key Steps to Transition From Low- to High-Volume Pancreatic Surgery Center.

1. Standardize Patient Selection
a. Create a multidisciplinary team- fellowship trained leader
b. Present all patients at Tumor Boards-
c. Provide continuity and oversight of patients undergoing pancreatic surgery
2. Standardize perioperative protocols, such as
a. Enhanced Recovery After Surgery (ERAS)
b. Operating room procedures for anticipated high-acuity cases, and
c. Align postoperative intensive care
3. Standardize review of outcomes
a. Objective classification of events using Clavien Dindo system
b. Objective classification of pancreatic-specific complications using the International Study Group of Pancreatic Surgery standards
c. Presentation of all complications at peer review conferences
d. Validation of local data using the National Surgical Quality Improvement Program (targeted) for risk adjusted outcomes

We monitored postoperative complications using the Clavien-Dindo [3] classification, peer review and pancreatic-specific complications using the International Study Group Pancreatic Surgery (ISGPS) guidelines [4]. An internal database was created with institutional review board approval for collection of patient data. Risk adjusted outcomes were validated using the targeted pancreatic database the American College of Surgeons National Quality Improvement Program [5].

## RESULTS

We prospectively followed a total of 119 patients who underwent pancreatic surgery between September 2016 to January 2020. Overall, 48.7% were male, and mean ± SD age was 65.3 ± 12 years. We noted a diverse patient population, reflecting our local heterogeneous community -the majority of patients were African American (40.8%), followed by East Asian or Pacific Islander (23.3%), and Caucasian (19.4%). 17.6% identified as Hispanic ethnicity. Comorbidities were also common, including hypertension (70.6%) and diabetes mellitus (40.3%). After implementation of our program in 2017 our percent of acute renal failure requiring hemodialysis also increased. The average American Society of Anesthesia (ASA) physical status classification was 2.6 ± 0.54 for all patients.

During the 2 years prior to creating a dedicated service, 16 patients underwent pancreatic surgery at our institution. In the following 3 years an additional 103 patients underwent surgery. In 2020, the SARS-CoV-2 pandemic decreased the volumes but the program recovered in 2021 with 34 resections.

After creating the dedicated program, tumor board presentations markedly increased from 20 per quarter to over 120 per quarter, with over 50% hepatico-pancreatico-biliary. This expansion resulted in increased frequency of tumor board and more complex gastrointestinal conditions.

With the HPB team in place, 50.2% resections were classic pancreaticoduodenectomy, 9.7% were pylorus-preserving, 26.2% distal pancreatectomy (mostly laparoscopic or robotic), 3.9% central and 5% total pancreatectomy. Vein resections were performed in 5.8% of patients. Pancreas texture was noted to be soft in 71.8%, while in contrast, duct size was more variable, with 54% patients with a duct size < 3 mm, 22.3% with a size 3-6 mm, and the rest greater than 6 mm. Pancreatic fistulas developed in 17 (16.5%) patients, of which 11 were Grade B. Delayed gastric emptying was seen in (9.7%) patients, all of whom had undergone pancreaticoduodenectomy. While these complications did not differ from the low-volume time period, after creation of the dedicated service the average hospital length of stay decreased from 14.1 ± 6.3 days to 10.8 ± 5.8 days in 2020. The 30-day mortality was 2.9% of patients during the high-volume period.

When compared to calendar year 2017, the risk-adjusted semi-annual report for NSQIP for calendar year 2019 confirmed a safe transition. There was no change in Whipple mortality (Odds Ratio 0.93 vs 0.97), morbidity (1.12 vs 1.24) and fistula rate (0.70 vs 0.77). There was a decrease in delayed gastric emptying (1.17 vs 0.99) cardiac events (1.08 vs 0.83) and sepsis (1.63 vs 1.01) and an increase in unplanned intubation (0.97 vs 1.11) and pneumonia (0.88 vs 1.26). Interventions to improve these parameters worked at the expense of increasing length of stay such that our current 2020 report shows a "need to improve" in that field.

## CONCLUSION

We have shown that using 4 steps (Table 1) a hospital can rapidly and safely transition from a low volume service to high volume pancreatic center. Importantly, our on-site database outcomes were validated using the risk-adjusted targeted NSQIP program. While we show the program to be successful for pancreatic surgery, these patient and outcome-oriented steps, using widely accepted standardized tools, are adaptable to other high risk procedures as well.

While the centralization of pancreatic surgery has many benefits [6], equitable access to these centers remains difficult for underserved populations. African American and Hispanic patients have lower rates of surgical resection and are more likely to be treated at low-volume pancreatic surgery hospitals [7], and this is associated with worse postoperative survival [8]. Our experience underscores how, using the steps outlined in the Table 1, dedicated physicians can bring complex surgeries safely to appropriate hospitals in otherwise-underrepresented communities.

## Disclosures

### Author contributions

Conception and Design: Drs Wayne, Zenilman, Steele, Fahoum.

Data Collection, analysis, interpretation: Dr. Cho, Zenilman, Wayne, McClelland, Rodriquez.

Drafting of manuscript: Dr. Cho, McClelland, Zenilman.

Critical Review of manuscript: Dr. Cho, Zenilman, Wayne.

Final approval of version: Drs Cho, Wayne, Zenilman, McClelland, Fahoum, Steele, Fahoum.

## Funding sources

The authors report no funding sources for this project.

## Ethics approval statement

Internal institutional review board approval was obtained prior to collection of patient data.

## Conflicts of interest

The authors report no conflicts of interest.
